# The Transcranial Cisternal Port (TCP): A Novel Technique for Repetitive Intrathecal Delivery in Mice

**DOI:** 10.21203/rs.3.rs-8080141/v1

**Published:** 2025-12-01

**Authors:** Benedikt E. Haupt, Jillyn Turunen, Ian Olson, Atique U. Ahmed, Irina V. Balyasnikova

**Affiliations:** Northwestern University; Northwestern University; Northwestern University; Northwestern University; Northwestern University

**Keywords:** Intrathecal delivery, Cisterna magna, Immunotherapy, CSF dynamics

## Abstract

**Purpose:**

Cerebrospinal fluid (CSF)-directed therapies are gaining importance in neuro-oncology, but long-term CSF access in preclinical murine models remains limited by anatomical constraints. The Transcranial Cisternal Port (TCP) is a skull-anchored cannula that provides repeated access to the murine intrathecal space.

**Methods:**

The procedure is performed by: making an incision over the craniocervical junction and exposing the cisterna magna; performing a craniostomy on the interparietal bone and placing a guide cannula through it; verifying placement by threading a wire through the cannula and visualizing it within the cisterna magna; securing the cannula in place using surgical adhesive; and closing skin and inserting a mandrin to maintain patency. The TCP was validated via Evans blue dye injection, and long-term outcomes were assessed over 3 weeks in 43 athymic nude mice.

**Results:**

Evans blue dye distribution was observed throughout the cisterna magna, basal cisterns, and perivascular spaces. All animals recovered uneventfully without device-related complications. Functional retention was 93% at day 7, 86% at day 14, and 86% at day 21. Failure was related to intraluminal clogging (n = 4) and adhesive bonding of the dummy to the guide cannula (n = 2). Revision surgery was successful in all cases.

**Conclusion:**

The TCP provides a skull-anchored access route to the murine CSF, enabling reliable intrathecal delivery. Cannula patency remains a limitation in some animals. Future iterations might mitigate this by improving guide cannula design. Overall, these findings establish the TCP as a scalable, functionally robust platform for modeling intrathecal regimens in preclinical studies.

## 1. INTRODUCTION

Delivery of systemic drugs to the central nervous system (CNS) is limited by the blood–brain barrier, blood–tumor barrier, and blood–cerebrospinal fluid barrier. Intrathecal administration offers a direct route to the CNS by circumventing these barriers [1]. The intrathecal space is formed by the meningeal coverings of the CNS that hold cerebrospinal fluid (CSF). Various catheter-based systems have been developed to maintain extended intrathecal access [2–8]. There is a growing need for preclinical models to evolve accordingly: they should provide anatomically accurate, scalable, and clinically relevant methods of delivery that mirror the approaches used in patient care. While single intrathecal injections are commonly used and relatively well established in preclinical models, extended access for repeated therapeutic delivery remains technically challenging. The small anatomical dimensions of the murine CNS impose significant constraints. In addition, many existing techniques lack reproducibility, scalability, or real-time confirmation of correct placement. Thus, they often fall short when applied in real-world experimental workflows.

The cisterna magna (cerebellomedullary cistern) is a subarachnoid cistern situated between the dorsal medulla and the inferior surface of the cerebellum. It is covered by translucent meningeal membranes which provide a natural window that allows direct visualization of the intrathecal space. The cisterna magna has been extensively used to study the glymphatic system [9], as well as for intrathecal therapeutic delivery [10] and for establishing leptomeningeal disease models [11, 12]. Despite these applications, existing cisterna magna-based techniques are primarily limited to single-use injections [13], and no existing method allows for scalable and reproducible cisternal access over extended periods.

Here, we present the Transcranial Cisternal Port (TCP), a novel technique with a rapid surgical workflow enabling visually guided and repeated access to the cisterna magna via a cranially anchored guide cannula.

## 2. MATERIALS AND METHODS

### 2.1 Animals

Healthy 6–8-week-old male and female C57/B6 and athymic nude mice were utilized (The Jackson Laboratory). Animals were euthanized in accordance with institutional guidelines using CO_2_ inhalation followed by cervical dislocation. Northwestern University Institutional Animal Care and Use Committees approved animal experiments.

### 2.2 Materials

- Stereotactic frame (Stoelting)
- Stereomicroscope (Zeiss)
- Microsurgical scissors
- Microjeweler’s forceps, curved and straight tip
- Cotton-tip applicators
- High-speed surgical drill with cutting burr (1mm)
- 25 μL Hamilton microsyringe (Hamilton Company) fitted with a 33-gauge, 1.5 cm stainless steel needle (straight shaft, flat bevel)
- Cyanoacrylate surgical adhesive
- 26-gauge stainless steel guide cannula (C315GS-5/SPC, Protech International Inc.; overall length ~ 11 mm, length below pedestal: 2 mm)
- Matching screw-on Mandrin with 0.5 mm projection (C315DCS-5/SPC, Protech International Inc.) (also called “dummy cannula”)

### 2.3 Surgical Protocol

#### 2.3.1 Animal Preparation

**Figure 1.**
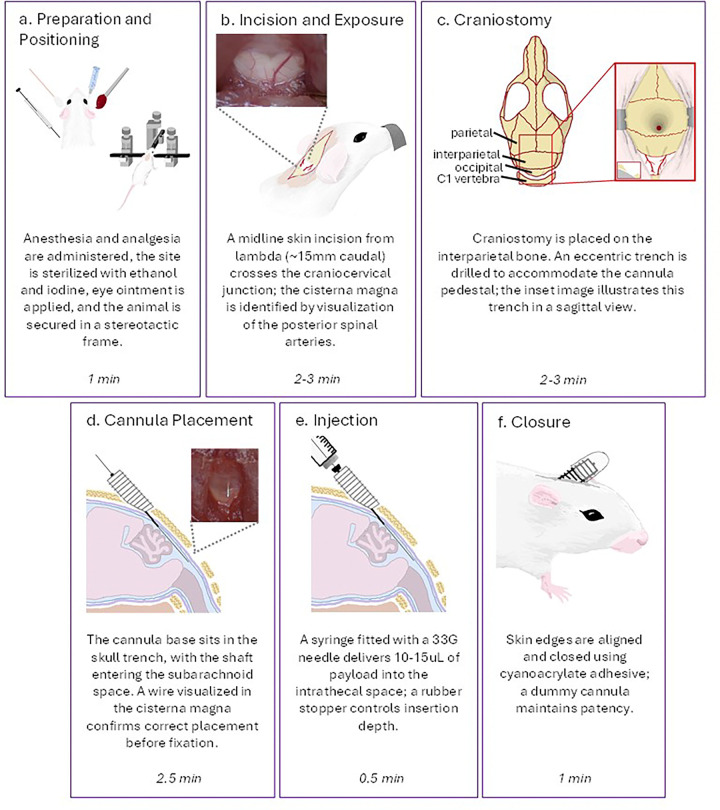
Surgical placement of the Transcranial Cisternal Port (TCP). (a) Anesthesia and analgesia are administered, the site is sterilized with ethanol and iodine, eye ointment is applied, and the animal is secured in a stereotactic frame. (b) A midline incision from lambda (~15 mm caudal) crosses the craniocervical junction; the cisterna magna is identified by visualizing posterior spinal arteries. (c) A craniostomy and an eccentric trench are drilled on the interparietal bone to accommodate the cannula pedestal (shown in sagittal view in inset image). (d) The cannula base sits in the skull trench with the shaft traversing the subarachnoid space. A wire seen in the cisterna magna confirms correct placement before fixation. (e) A syringe fitted with a 33G needle delivers 10–15 μL of payload into the intrathecal space; a rubber stopper controls insertion depth. (f) Skin closure with cyanoacrylate; a dummy cannula maintains patency. Figure created in Microsoft Paint

Instruments are sterilized via autoclave before beginning the procedure. Mice are anesthetized with ketamine-xylazine in accordance with institutional guidelines. Anesthesia is confirmed with loss of the foot pinch reflexes bilaterally. The surgical field is prepared by cleaning the skin with ethanol and iodine swabs, followed by sterile draping. Ophthalmic lubricant is applied to both eyes to prevent corneal drying during anesthesia (Fig. 1a).

#### 2.3.2 Animal Positioning

The animal is placed in a stereotactic frame using ear bars and an incisor clamp to achieve stable three-point fixation (Fig. 1a). Once the skull is secured, the incisor clamp is lowered approximately 5 mm to incline the head and expose the craniocervical junction. This adjustment creates tension across the overlying membrane to facilitate cisterna magna visualization and access, but overextension must be avoided to prevent cervical injury.

#### 2.3.3 Incision and Exposure of the Cisterna Magna

All steps are performed under a stereomicroscope using a standard microsurgical technique. A midline skin incision starts at lambda and extends approximately 15mm caudally over the craniocervical junction. Blunt dissection is then performed along the midline using cotton-tip applicators and fine microforceps, following the plane of the nuchal ligament. The muscle is separated atraumatically, preserving its insertions. This exposes the translucent meningeal membranes and the underlying cisterna magna, in which the posterior spinal arteries are visualized (Fig. 1b).

#### 2.3.4 Craniostomy and Cannula Bed Preparation

The periosteal membrane is gently removed, and the exposed skull surface is dried using a sterile cotton swab. A high-speed surgical drill with a fine-cutting burr is then positioned at the midline, approximately 1 mm posterior to lambda on the interparietal bone. A small craniostomy is created by carefully drilling through the outer and inner tables of the skull until the underlying dura becomes visible. To accommodate the guide cannula base, a shallow semicircular trench is milled around the anterior edge of the craniostomy without fully penetrating the cranial bone. This creates an eccentric, bowl-shaped depression that smooths the bony margins and provides a stable, flush seating surface for the cannula pedestal (Fig. 1c).

#### 2.3.5 Implantation of the Guide Cannula

Using a sterile scalpel, the plastic base of the cannula is trimmed at approximately 45°, allowing the shaft below the pedestal to enter the skull at an angle while the pedestal remains seated within the prepared bone depression. This adjustment facilitates an optimal midline trajectory toward the cisterna magna (Fig. 1d). A small drop of surgical cyanoacrylate adhesive is applied to the anterior portion of the cannula pedestal to secure it in place temporarily. The trajectory and insertion depth are verified using a thin wire (approximately 35G). The wire is inserted through the guide cannula until it becomes visible within the center of the cisterna magna. To determine the required injection depth, the proximal end of the wire is grasped with fine forceps at the point where it exits the cannula, and the length is measured against a millimeter scale. In our setup, the optimal insertion depth is 11 mm. There is sufficient working time before the adhesive fully hardens, allowing this step to be performed without haste. Once correct positioning and depth are confirmed, the remaining cyanoacrylate is circumferentially applied around the pedestal to secure the cannula to the skull permanently.

#### 2.3.6 Optional Intrathecal Injection

A 25 μL Hamilton syringe fitted with a 33G needle is loaded with 10–15 μL of the injection solution. A depth-limiting collar (i.e., rubber stopper) is adjusted to the predetermined insertion depth. The needle is then inserted through the guide cannula, and the payload is delivered slowly at approximately 1 μL per second (Fig. 1e). After injection, the needle is left in place for an additional 10 seconds to allow pressure equilibration and to minimize reflux along the needle tract upon withdrawal.

#### 2.3.7 Muscle Reapproximation, Skin Closure, and Dummy Cannula Insertion

The muscle layer is reapproximated carefully without tension. The skin edges are aligned and closed with a thin layer of cyanoacrylate adhesive. After the adhesive has completely dried, the dummy cannula is screwed into place (Fig. 1f). Care must be taken to ensure that the adhesive is fully cured before inserting the dummy cannula to avoid accidental bonding to the internal threads of the guide cannula.

#### 2.3.8 Post-Surgical Care

Postoperative analgesia is provided using meloxicam and buprenorphine, administered before recovery, and continued as needed. Postoperative anesthesia reversal is performed, and then mice are placed in warmed recovery cages and observed continuously until full ambulation is regained. Standard chow and water are provided ad libitum. Analgesia, anesthesia reversal, and supportive care are administered in accordance with institutional guidelines.

#### 2.3.9 Reinjection Procedure

For intrathecal access subsequent to cannula placement surgery, the mouse is re-anesthetized. The dummy cannula is removed, and the animal is positioned on a flat surface with a slight head inclination. Alternatively, the mouse can be positioned in a stereotactic frame, as described in Section 2.3.2. The injection procedure is then repeated following the protocol outlined in Section 2.3.6.

## 3. RESULTS

### 3.1. Confirmation of Intrathecal Delivery

**Figure 2.**
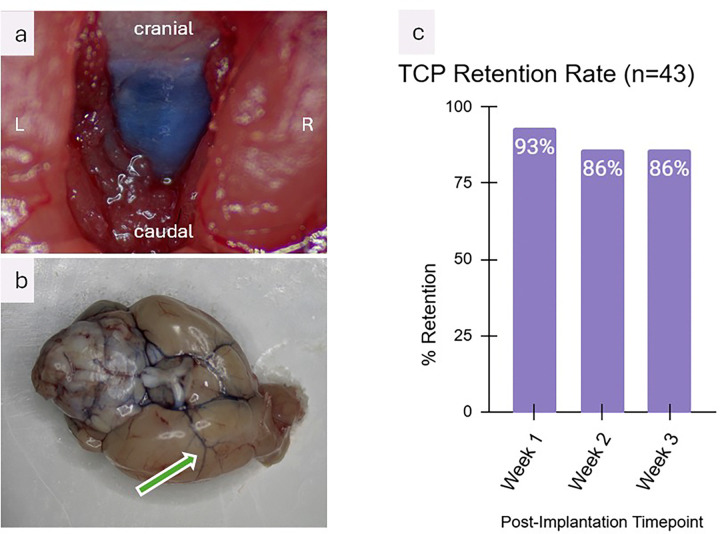
Validation of the Transcranial Cisternal Port (TCP). (a) Intraoperative microscopic view showing the exposed cisterna magna following injection of 15 μL Evans Blue via the TCP; there is robust dye distribution throughout the cisterna magna. (b) The basal surface of the murine brain, post-Evans Blue injection, demonstrates dye distribution across the basal cisterns and along perivascular spaces (arrow). (c) Functional retention of the TCP implant was assessed longitudinally, with retention rates of 93% at 1 week and 86% at both 2 and 3 weeks post-implantation (n = 43)

To confirm that injections via the TCP reached the CSF compartment, 15 μL of Evans blue dye was injected as described in Section 2.3.6 (Fig. 1e). Successful visualization of the needle tip within the cisterna magna was achieved, and distribution of the dye within the cisterna magna was observed; a representative image is shown in Fig. 2a. After 5 minutes, the animal was sacrificed, and the brain was harvested. As shown in Fig. 2b, the dye was distributed throughout the basal cisterns, and penetration into perivascular spaces was clearly observed under higher magnification (arrow).

### 3.2 Long-Term Neurological Follow-Up and Functional Cannula Retention

To assess the technical reliability and functional retention of the TCP under conditions of high-frequency manipulation, 43 athymic nude mice were implanted with a TCP and followed over 3 weeks. Although a leptomeningeal tumor model was established via the TCP on the day of implantation, with mice receiving weekly intrathecal injections and undergoing weekly bioluminescent imaging, therapeutic and oncologic outcomes fall outside the scope of this study and will be presented separately.

Mice were group-housed, and post-implantation recovery was universally uneventful: all animals regained full neurological function without signs of ataxia, focal deficits, weight loss, CSF leakage, or wound complications. Neurological deficits began to emerge beyond day 21 due to tumor burden and were unrelated to the cannula system.

Functional retention was defined as cannulas remaining patent and injectable. At post-implantation day 7, the functional retention rate was 93% (40/43). Losses were due to one irreversibly blocked cannula and two cases where the dummy became permanently bonded to the guide cannula, necessitating removal. On day 14, three additional cannulas were deemed nonfunctional due to blockage, resulting in a retention rate of 86% (37/43), which remained stable at day 21 (Fig. 2c).

Beyond 21 days, cohort-wide survival declined due to disease burden, precluding reliable retention statistics past this time point. The maximum observed retention was 58 days in a small subset (n = 2), after which animals were sacrificed for non-cannula-related reasons.

### 3.3 Technical Observations and Efficiency Considerations

**Figure 3.**
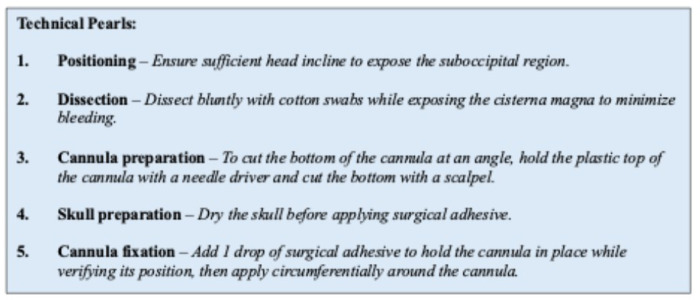
Technical pearls

Several procedural factors were identified as critical for effective intrathecal delivery and are summarized in Fig. 3. For instance, precise angulation of the cannula is essential to avoid impingement on the inner table of the occipital bone, as a too-shallow trajectory can result in insufficient insertion depth. The verification wire should be left in place until the adhesive is fully cured to avoid cannula displacement during fixation, ensuring the intended trajectory is maintained.

Once optimized, the procedure requires approximately 10 minutes per mouse and can be performed by a single surgeon with the support of one assistant. This allows for TCP placement in up to 40 mice in a single 6-hour working session.

The surgical steps are as follows: stereotactic positioning requires approximately 1 minute once mice are anesthetized. Exposure of the cisterna magna typically takes 2–3 minutes, followed by preparation of the cannula bed, which requires an additional 2–3 minutes. Trajectory and, if needed, depth verification using a guide wire takes approximately 30 seconds. Final fixation of the cannula requires another 2 minutes. The optional injection itself may take 30 seconds. Final skin closure takes around 1 minute (Fig. 1a–f).

### 3.4 Revision Surgery for Functional Cannula Loss

Reimplantation was attempted in animals with functional cannula loss during the postoperative period. As expected with revision surgeries, moderate bleeding was consistently observed—typically diffuse, persistent oozing from scarred subcutaneous layers. The bleeding was best managed with gentle but sustained pressure using sterile cotton swabs. In most cases, sharp dissection was only necessary to reopen the skin; underlying layers could be separated by blunt dissection along the original surgical plane.

The craniostomy site often exhibited fibrous scar coverage. This could be easily cleared using a brief pass of a high-speed surgical drill to re-establish a clean surface for proper cannula seating. As in the primary surgery, complete skull drying was essential to ensure cyanoacrylate adhesion. Cannula reimplantation then proceeded as previously described.

## 4. DISCUSSION

The current landscape of indwelling intrathecal catheter models presents several critical challenges which the Transcranial Cisternal Port (TCP) was designed to address. The overarching goal is to replicate the functionality of clinical intrathecal systems, such as the Ommaya reservoir, to enable repeated therapeutic delivery over a treatment cycle, including in large experimental cohorts.

A major challenge of murine intrathecal delivery systems is the lack of reliable confirmation of cannula placement. Unlike in clinical settings, where CSF backflow, fluoroscopy, or direct intraoperative visualization confirm placement, most murine methods depend on indirect proxies which offer little assurance of actual intrathecal access. For instance, ventricular targeting in the murine Ommaya system relies on craniometric coordinates, which presupposes consistent anatomical landmarks and trajectory, but does not provide real-time feedback [14]. This presents a limitation in tumor-bearing models, where mass effect or peritumoral edema can distort ventricular anatomy, displace the cannula tip out of the intended compartment, or render standard coordinates unreliable. Although imaging modalities such as micro-CT or MRI can theoretically verify cannula placement, their cost, time requirements, and low throughput make them impractical for large-scale experiments involving dozens of animals. The cisterna magna offers a distinct advantage in this regard by serving as a natural anatomical window and allowing for direct visual confirmation of needle placement and distribution of the injected payload (Fig. 1e).

Establishing leptomeningeal disease and delivering therapeutics via single injections into the cisterna magna is relatively well established and has been described in detail elsewhere [12, 13, 15]. However, when transitioning from single-use injections to chronic, indwelling access ports, several approaches have been proposed, each with distinct technical limitations. While multiple surgical procedures can be performed serially, this approach raises concerns regarding animal welfare, increasing surgical difficulty due to scar tissue formation, and a fundamental lack of scalability.

Several reported methods rely on polyethylene (PE) tubing as the conduit for CSF access. For instance, Xavier et al. implanted PE tubing directly into the cisterna magna and secured it within the surrounding soft tissue using a combination of cyanoacrylate glue and dental cement [16].

However, this approach has several limitations. The catheter is embedded within soft tissue without anchoring to a rigid structure like the skull. Physiological movements of surrounding musculature or skin can generate shear forces at the catheter interface, increasing the likelihood of displacement and CSF leakage at the entry site. Additionally, only ~ 1–2 mm of the catheter resides within the cisterna magna, offering minimal mechanical reserve. Friedman et al. addressed some of these challenges in 1994 in a rat model by advancing a mandrin-guided PE-10 catheter through the atlanto-occipital membrane caudally along the spinal canal into the lumbar cistern, effectively bypassing the risk of displacement via deep intrathecal anchoring. However, replicating this method in mice would pose a substantial risk of spinal cord injury, given the significantly smaller subarachnoid space and more delicate anatomical proportions [17].

Catheter-based systems similar to the TCP have been described in rat models. Shapiro et al. adapted a technique initially described in 1983, placing PE tubing transcranially into the cisterna magna [18, 19]. Similarly, Hao et al. employed a PE tubing–based approach using a bent syringe needle as a penetrating stylet [20]. The overarching limitation across all PE tubing–based approaches is maintaining patency over time. Without a retained internal mandrin, the tubing is highly susceptible to clogging by blood, cellular debris, or proteinaceous CSF. Once occluded, restoring function is extremely difficult and often requires disruption of the surgical construct. Although PE tubing is frequently chosen for its perceived reduced risk of tissue trauma, this advantage is mainly theoretical. In practice, a metal stylet is still required—either during implantation, as seen in Friedman et al., or to maintain patency. Thus, the continued reliance on metal instrumentation negates the supposed benefit of a “soft” interface. Additionally, the dead space of PE tubing requires dosing adjustments and typically mandates a flush following each injection, introducing potential variability.

Unlike traditional catheter-based systems, where the conduit and injection interface are the same, the TCP deliberately separates structural access from the injection apparatus. The guide cannula serves solely as a stable, pre-aligned gateway to the CSF compartment, while the actual delivery is performed via an external injector (e.g., a Hamilton syringe with a depth-limited needle).

## 5. LIMITATIONS

Despite addressing several shortcomings of prior techniques, the TCP approach is not without limitations.

Functional cannula retention remains a technical challenge. The primary failure mode observed in our cohort (~ 10%) was irreversible clogging of the guide cannula, occurring in one case by day 7 and three by day 14 (Fig. 2c). This was most likely due to debris accumulation following injections of cellular and recombinant protein-based therapeutics, which are prone to aggregation within the cannula lumen. Crucially, the rigid, metallic architecture of the TCP system, unlike soft tubing-based platforms, allows for re-establishment of patency in most cases using controlled mechanical probing. Future iterations may incorporate guide cannulas with larger internal diameters, while maintaining fine-caliber injection needles, to increase the adequate clearance within the system, thereby increasing patency.

In addition to clogging, two cases of cannula dysfunction were attributed to adhesive bonding between the dummy cannula and the guide cannula, rendering them inseparable. This failure mode was specific to the adhesive used and likely resulted from delayed curing or capillary creep of the glue along the external thread of the guide. Careful control of curing time and application volume is essential to avoid this complication and ensure long-term dummy cannula removability.

The authors also observed that while CSF distribution can still occur with suboptimal needle placement, meaning within the subarachnoid space but outside the cisterna magna, distribution through the perivascular spaces was only visible when injections were delivered directly into the cisterna magna. This observation is supported by recent findings describing the subarachnoid lymphatic-like membrane, a fourth meningeal layer subdividing the subarachnoid space into superficial and deep compartments. These data suggest that the subarachnoid space is not a homogenous cavity but a structurally compartmentalized system that may influence solute dispersion depending on the injection site [21].

## 6. CONCLUSION

The Transcranial Cisternal Port (TCP) represents a skull-anchored gateway to the murine intrathecal space. Potential uses include establishing leptomeningeal disease models and administering intrathecal therapeutic regimens. The primary limitation of the current TCP system is the irreversible loss of patency due to luminal debris buildup. While reimplantation can partially mitigate this, future iterations incorporating improved cannula designs are expected to enhance long-term patency. Nonetheless, the present data establish a clear proof of concept: the TCP enables a rapid, scalable surgical workflow and, when functional retention is maintained, allows for efficient and reproducible intrathecal delivery over extended experimental timelines.

## Data Availability

All data generated or analysed during this study are included in this published article.

## Abbreviations

CNS: Central nervous system
CSF: Cerebrospinal fluid
PE: Polyethylene
TCP: Transcranial Cisternal Port
